# Identifying Key Bioactive Components in Postbiotic Preparations: From Candidate Discovery to Functional Validation

**DOI:** 10.3390/foods15162893

**Published:** 2026-08-18

**Authors:** Qingqing Yu, Mengting Liu, Yansheng Zhao, Xiang Xiao

**Affiliations:** School of Food Science and Engineering, Jiangsu University, Zhenjiang 212013, China; 18855094424@163.com (Q.Y.); liumengting2000@163.com (M.L.); zhaoys@ujs.edu.cn (Y.Z.)

**Keywords:** postbiotics, bioactive components, evidence appraisal, functional attribution, multi-omics

## Abstract

Postbiotic preparations contain inanimate microbial cells and a diverse mixture of cell-wall structures, proteins, polysaccharides, lipids, metabolites, and vesicle-associated materials. Although many of these components have been proposed as key bioactives, most are identified through compositional profiling, enrichment in active fractions, or testing of purified molecules. Such findings demonstrate biological activity but do not necessarily show that a candidate contributes to the effect of the original preparation. This review examines the experimental approaches used to narrow candidate lists and evaluate functional contribution, including phenotype-guided comparison, activity-guided fractionation, selective depletion, multi-omics profiling, structural characterization, dose–response testing, mechanistic intervention, and reconstitution. A structured narrative search of Web of Science Core Collection, PubMed, and Scopus through 30 March 2026, supplemented by citation tracking, identified the relevant literature; 15 representative primary studies that examined defined candidates and provided evidence beyond compositional detection were selected and appraised across seven preparation-level attribution domains. An appraisal of representative studies shows that current evidence largely supports the activity of individual candidates, whereas preparation-level attribution remains uncommon. Stronger evidence requires quantification of the candidate in the source material, selective removal with appropriate controls, and restoration at a preparation-relevant dose. Structural heterogeneity, processing history, molecular state, dose, and experimental context must also be considered. This evidence-based approach can support bioactive-component validation, batch consistency, and the design of future preclinical and human studies.

## 1. Introduction

Postbiotics have attracted increasing attention as functional preparations because of their potential advantages in stability, safety, and processing compatibility [1,2]. Yet the expansion of postbiotic research has exposed a more fundamental problem: beneficial effects are increasingly reported for complex preparations, whereas the components that produce those effects are seldom established directly [3,4,5]. The field therefore needs to distinguish candidate discovery from causal attribution [6,7,8].

Postbiotic preparations have been associated with immunomodulatory, barrier-protective, antimicrobial, and metabolic effects, and several studies have begun to move from whole-preparation phenotypes toward the identification of specific microbial effectors [9]. Pasteurized *Akkermansia muciniphila* provides a frequently cited example. Its outer-membrane protein Amuc_1100 reproduced several barrier-related and metabolic effects associated with the pasteurized bacterium and was linked to TLR2-related signaling [10]. Such experiments establish that a purified molecule can be active, but not necessarily that it accounts for the effect of the source preparation. That stronger claim requires experiments conducted in, or quantitatively anchored to, the preparation itself.

The distinction between candidate-specific activity and preparation-level contribution is particularly important when interpreting the screening strategies commonly used in postbiotic research. Activity-guided fractionation and metabolomic, proteomic, or lipidomic profiling can localize activity and rank molecular candidates, but differential abundance is not equivalent to functional necessity. A feature may increase because processing releases it, because it covaries with another constituent, or because it becomes easier to detect. This distinction is especially important for postbiotics, in which inanimate cells, envelope structures, soluble metabolites, extracellular vesicles, and lysis-derived material can coexist. Their relative abundance and accessibility depend on strain, cultivation, inactivation, downstream processing, storage, and the food matrix [1,11]. The key question is therefore not only whether an isolated microbial molecule is biologically active, but whether it contributes to the activity of the postbiotic preparation in which it occurs.

Most existing reviews have mainly focused on definitions, functions, composition, or applications. However, less attention has been given to what different experimental designs can establish about the contribution of a specific component to the activity of its parental preparation. This review therefore examines the progression from candidate discovery to functional validation by evaluating the strengths, limitations, essential controls, and common sources of misinterpretation associated with phenotype-guided comparison, fractionation, selective depletion, multi-omics profiling, structural characterization, reconstitution, dose–response analysis, receptor intervention, and cross-model validation. A structured evidence scheme is used to distinguish proposed candidates, activity-associated candidates, class-level contribution, candidate-specific functional support, and preparation-anchored contribution. These categories describe the type and extent of available evidence rather than a numerical hierarchy.

### Literature Search and Study Selection

This structured narrative review searched Web of Science Core Collection, PubMed, and Scopus from database inception to 30 March 2026. Search terms combined descriptions of postbiotic preparations (“postbiotic,” “inanimate microorganism,” “non-viable,” “heat-killed,” “pasteurized,” and “cell-free supernatant”) with candidate-component terms (“protein,” “peptide,” “polysaccharide,” “lipoteichoic acid,” “peptidoglycan,” “lipid,” “metabolite,” and “extracellular vesicle”) and functional-evaluation terms (“fractionation,” “depletion,” “add-back,” “validation,” and “mechanism”). Reference lists and forward citations were also examined. Only peer-reviewed articles published in English were considered.

For the evidence-appraisal table, 15 primary studies were selected when they examined a defined candidate molecule, component class, or molecular feature and included evidence beyond compositional detection, such as fractionation, candidate testing, depletion or add-back, dose–response analysis, mechanistic intervention, or in vivo validation. The selected studies covered the major component classes and evidence categories discussed in this review. When several studies provided similar evidence, the study with the clearest experimental design and reporting was retained. A few boundary examples that did not fully meet the ISAPP definition were included to clarify the difference between the activity of an isolated microbial molecule and its contribution to a defined postbiotic preparation. The table is therefore representative rather than exhaustive and should not be used to estimate the prevalence of each evidence type in the literature.

## 2. Defining Postbiotics and the Boundaries of Bioactive-Component Research

### 2.1. Defining Postbiotics and Their Boundaries

A clear definition of postbiotics is needed before their bioactive components can be discussed. The 2021 ISAPP consensus defined a postbiotic as “a preparation of inanimate microorganisms and/or their components that confers a health benefit on the host” [1]. Under this definition, the term refers to a preparation rather than to any substance produced by microorganisms. Inanimate microbial cells or their structural components form the basis of the preparation, while metabolites and other materials retained or released during processing may also be present. This distinguishes postbiotics from the much broader group of microbial products and metabolites.

This point is particularly important in bioactive-component research. Microbial metabolites such as short-chain fatty acids, organic acids, bacteriocins, and exopolysaccharides may contribute to host regulation, but microbial origin alone is not sufficient to classify them as postbiotics [1]. Likewise, purified single compounds with demonstrated activity are better regarded as postbiotic-derived candidate bioactive components or effector molecules than as postbiotics themselves [9]. Accordingly, the evidence discussed in this review is separated into three groups: direct postbiotic evidence from preparations meeting the ISAPP definition; postbiotic-derived component evidence from molecules isolated from or quantitatively linked to a defined postbiotic preparation; and methodological or mechanistic analogues, including culture supernatants, secretomes, live-cell mutants, and defined bacterial ligands. Analogue studies may inform candidate discovery or mechanisms, but they cannot directly establish contribution to a defined postbiotic preparation. These evidence-source groups are distinct from the functional-attribution categories used later in this review.

### 2.2. Major Sources of Postbiotic Bioactive Components

Postbiotic bioactive components may originate from three main sources: cell wall and surface-associated structures, extracellular or soluble factors, and intracellular or lysis-derived constituents released during inactivation or cell disruption [12]. Representative components include peptidoglycan (PGN), wall teichoic acid (WTA), lipoteichoic acid (LTA), surface proteins, membrane lipids, short-chain fatty acids (SCFAs), organic acids, peptides, bacteriocins, exopolysaccharides (EPS), enzymes, nucleic acid fragments, and other released cellular products [13]. These three groups are based mainly on where the components originate in relation to the microbial cell. Cell wall- and surface-associated molecules may remain attached to the cells, but they can also become exposed or released during processing. Extracellular components are found mainly in the surrounding medium, whereas intracellular components are released when membrane integrity is disrupted or the cells are lysed. Processing may therefore change whether a component is present in the cell-associated or soluble fraction. Its location in the final preparation, as well as its cellular origin, should be considered when assigning it to a particular group.

The amount and type of evidence available also differ among component classes. Cell envelope- and surface-associated structures, including lipoteichoic acids, peptidoglycan-related molecules, and certain surface proteins, have received relatively extensive attention because their cellular location is clear and many are involved in host recognition [12,13]. By contrast, soluble and lysis-derived materials include chemically diverse molecules whose abundance and accessibility may vary with cultivation and processing conditions. This source-based classification is useful for organizing candidate discovery, but it does not by itself establish which component contributes to the biological activity of the complete preparation.

### 2.3. Why Functional Attribution Is Difficult in Postbiotics

The main challenge in postbiotic bioactive-component research is not the detection of candidate molecules itself, but establishing whether, and to what extent, a candidate contributes to the biological activity of a complex preparation. Postbiotics are intrinsically multicomponent systems in which structural components, soluble metabolites, and lysis-derived products may coexist and act independently or jointly [9,14]. The activity of such a preparation may arise from a dominant component, the additive effects of several constituents, or interactions that are lost when the constituents are isolated. An active fraction or a molecular feature associated with a strong phenotype can therefore provide a useful lead, but it should not automatically be treated as the functional driver of that phenotype. Even for well-studied cell envelope- and surface-associated structures, direct evidence linking a specific component to the activity of the parental postbiotic preparation is often incomplete. For soluble and lysis-derived candidates, attribution can be further complicated by extraction conditions, purity, dose, and the coexistence of other active constituents.

This attribution is complicated further because the composition of a postbiotic is partly determined during its manufacture. Strain background, culture conditions, inactivation methods, and downstream processing can all alter the retention, exposure, release, or transformation of candidate components. Preparations produced from the same strain may consequently differ in both composition and biological activity [15,16,17]. Activity may also depend on fine structural features, such as polysaccharide architecture, lipoteichoic acid composition, or protein conformation, rather than on component class or abundance alone [13,18]. A polysaccharide-rich or protein-rich fraction, for example, may show activity even though only a particular structural form within that fraction is responsible for the response. The same component may also behave differently across experimental systems because its effects depend on receptor expression, the intestinal microenvironment, inflammatory status, dose, route of exposure, and the endpoint being measured [19].

Variation in preparation and testing conditions makes comparisons across studies difficult and has slowed the development of a common basis for postbiotic dose standardization. Unlike viable probiotics, which can often be quantified using colony-forming units, postbiotics cannot be adequately described by a single measure that captures their composition and activity [20]. Although the same basic questions apply to different candidates, their presence, abundance, functional contribution, and mechanism, the methods required to answer them vary among proteins, polysaccharides, cell-wall components, metabolites, lipids, and extracellular vesicles. Research should therefore move beyond candidate detection and association toward experiments that directly test selected components within the original preparation [5].

## 3. Molecular Basis of Postbiotic Bioactive Components

**Figure 1:**
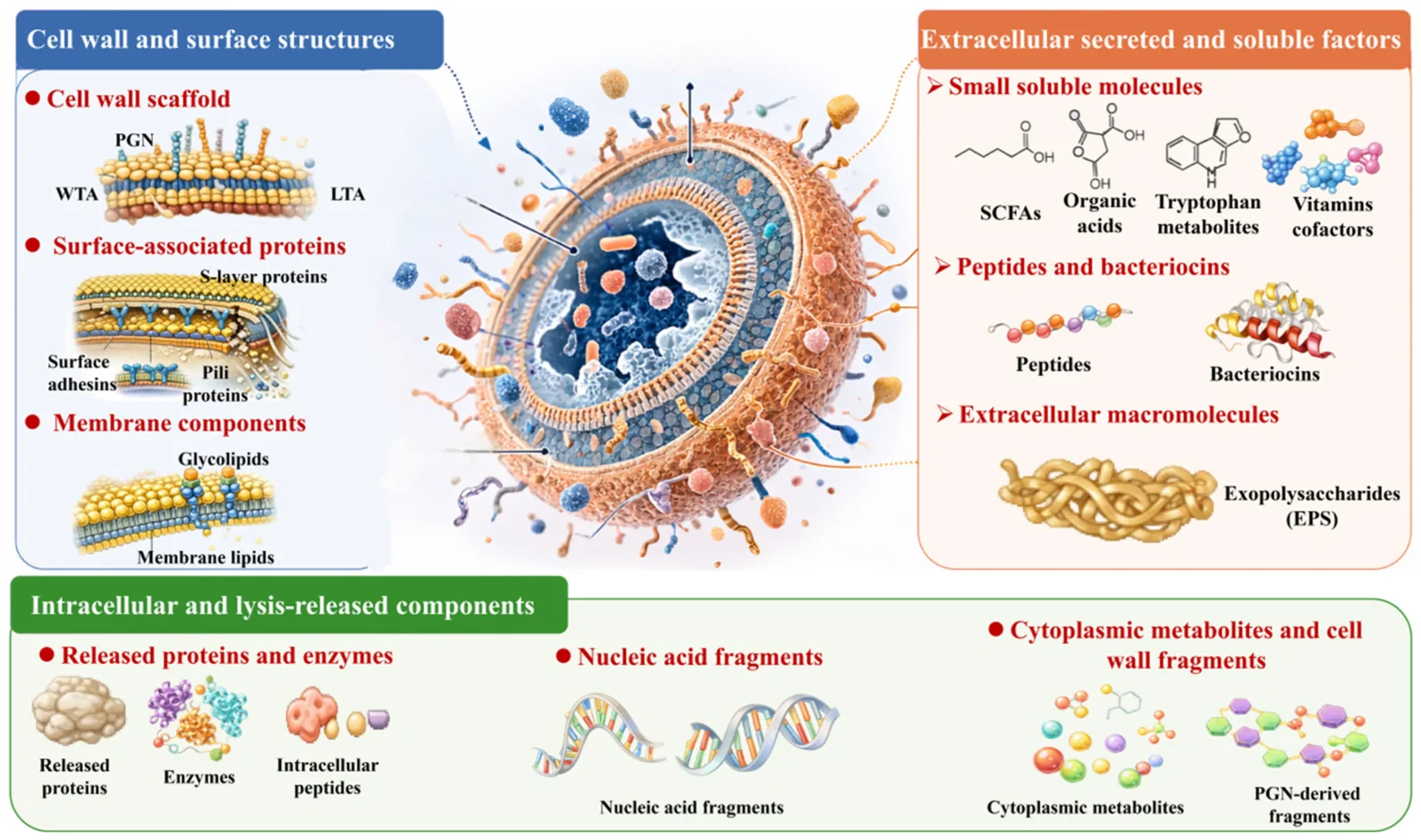
Major sources of candidate bioactive components in postbiotic preparations. Candidate bioactive components can be grouped into three main source categories: (1) cell wall and surface structures (e.g., PGN, WTA, LTA, surface proteins, and membrane lipids); (2) extracellular secreted and soluble factors (e.g., SCFAs, organic acids, peptides, bacteriocins, and EPS); and (3) intracellular and lysis-released components (e.g., proteins, enzymes, nucleic acid fragments, and cytoplasmic metabolites). The blue, orange, and green sections represent cell wall and surface structures, extracellular secreted and soluble factors, and intracellular and lysis-released components, respectively. Abbreviations: PGN, peptidoglycan; WTA, wall teichoic acid; LTA, lipoteichoic acid; SCFAs, short-chain fatty acids; EPS, exopolysaccharides.

Candidate components differ in location, accessibility, structural heterogeneity, and amenability to selective intervention (Figure 1). These differences determine how confidently a measured feature can be linked to biological activity. The sections below therefore compare cell-envelope structures, extracellular soluble factors, and intracellular or disruption-released material in terms of the specific attribution problems posed by each class.

### 3.1. Cell Wall and Surface-Associated Components

Cell wall and surface-associated structures include peptidoglycan, wall teichoic acid, lipoteichoic acid, surface-layer proteins, adhesins, pilus-related components, and membrane lipids or glycolipids [13]. Because these structures are located at the microbial envelope, their cellular origin and possible interactions with host receptors can generally be examined more directly than those of intracellular or lysis-derived constituents.

Peptidoglycan-derived muropeptides are recognized in a structure-dependent manner, with NOD1 and NOD2 responding to distinct peptide motifs [21]. Lipoteichoic acid has been linked to TLR2-dependent signaling [22]; removal or inactivation of LTA markedly reduced the macrophage-stimulating activity of supernatants from several Gram-positive bacteria [23]. Structural variation also influences the activity of surface proteins. S-layer proteins from *Lactobacillus kefiri* with different glycosylation patterns required different lectin partners for immunostimulation [24]. The effects of purified LTA from *Limosilactobacillus reuteri* L1 also varied with dose in cellular and mouse models [25]. These findings indicate that host responses depend not only on the general component class but also on molecular structure, dose, and experimental context.

This structural and experimental variability may account for some of the inconsistent effects reported for cell-envelope components. Lipoteichoic acid may induce inflammatory signaling in some systems but show immunomodulatory or barrier-protective effects in others, depending on its microbial source, purification procedure, dose, structural features, and the model used for testing. Similarly, the activity of a purified surface protein or lipid does not necessarily indicate that it is present in an accessible or functionally relevant form in the parental postbiotic preparation. Cell-envelope components are therefore reasonable candidates for mechanistic investigation, particularly when a corresponding receptor pathway can be identified. Nevertheless, their presence and receptor activity must be linked back to the preparation before they can be considered responsible for its overall biological effect.

### 3.2. Extracellular Secreted and Soluble Components

Extracellular secreted and soluble components include short-chain fatty acids, organic acids, tryptophan-derived metabolites, vitamins, bacteriocins, bioactive peptides, and exopolysaccharides [20]. These molecules are often highlighted in screening studies because they can be recovered from cell-free fractions and analyzed by metabolomic, proteomic, or chemical methods. However, this category includes both low-molecular-weight metabolites and larger macromolecules, which differ substantially in stability, purification, structural characterization, and biological exposure [26,27,28,29].

These components have been associated with antimicrobial, immunomodulatory, barrier-protective, and metabolic effects [30,31], but their contribution to a postbiotic preparation is often influenced by concentration, purity, culture-medium background, extraction conditions, and interactions with other constituents. Activity observed in an isolated or enriched fraction therefore does not necessarily establish that the same component is a major contributor to the parental preparation. Their functional relevance should be interpreted in relation to their actual abundance, structural state, and exposure within the original preparation [31].

### 3.3. Intracellular and Lysis-Derived Components

Intracellular and lysis-derived constituents are another source of candidate bioactive components in postbiotic preparations. Heat treatment, mechanical disruption, and other inactivation procedures can damage the cell envelope and release proteins, enzymes, peptides, nucleic acid fragments, and small molecules that would otherwise remain less accessible to the host [32,33,34,35]. Inactivation therefore changes not only microbial viability, but also the composition and accessibility of the final preparation.

However, the appearance of or increase in a component after processing does not necessarily indicate functional importance. It may result from release, degradation, or secondary transformation, and its contribution may vary with the extent of cell disruption and the processing conditions used. These factors make lysis-derived components difficult to compare across studies and highlight the need to distinguish processing-induced availability from demonstrated biological contribution.

### 3.4. Implications for Functional Attribution

The three source categories and their representative components are summarized in Table 1. Although this classification helps organize candidate components, it does not establish their contribution to the activity of the complete preparation. Their presence and accessibility also depend on cultivation, inactivation, and downstream processing, which can affect whether they are retained, exposed, released, or transformed. The following section therefore examines how these processing steps shape postbiotic composition and influence functional attribution.

## 4. Process-Related Factors Shaping Postbiotic Composition and Candidate Accessibility

**Figure 2:**
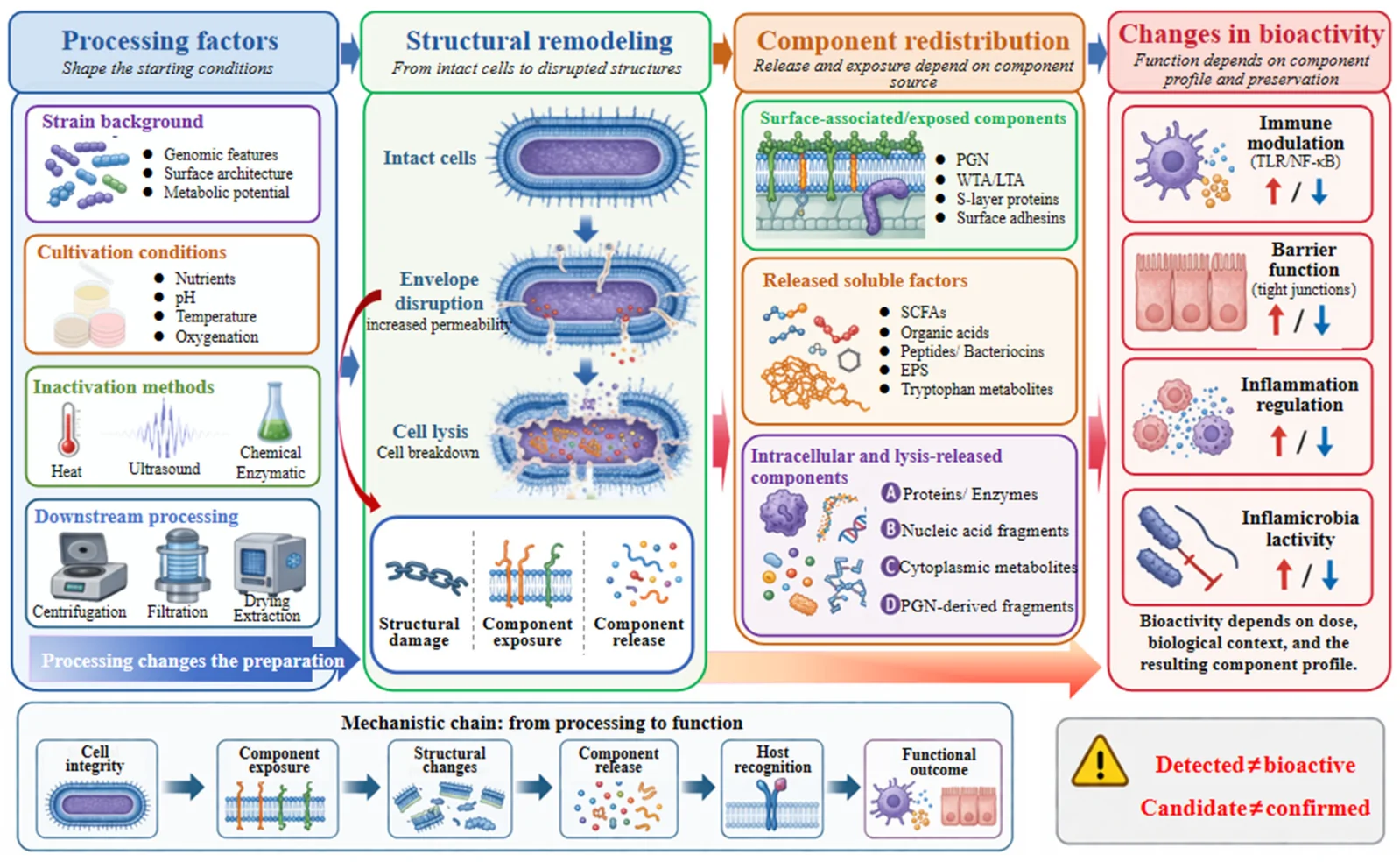
How strain background, cultivation conditions, inactivation methods, and downstream processing shape postbiotic component profiles and bioactivity. Blue, green, orange, and red panel frames represent processing factors, structural changing, component redistribution, and changes in bioactivity, respectively. Within the component redistribution panel, green, orange, and purple boxes denote surface-associated/exposed components, released soluble factors, and intracellular and lysis-released components, respectively. Horizontal arrows indicate the conceptual progression from processing factors through structural changing and component redistribution to changes in bioactivity, whereas the downward and curved arrows indicate progressive loss of cell integrity from intact cells to envelope disruption and cell lysis. In the bioactivity panel, red upward and blue downward arrows indicate potential increases and decreases in the indicated biological functions, respectively. The arrowed sequence at the bottom summarizes the proposed mechanistic chain from processing to functional outcome.

A postbiotic component profile is the product of a process history. Strain and cultivation determine the initial repertoire, whereas inactivation and downstream operations determine which molecules remain cell-associated, become exposed, enter the soluble phase, or undergo structural change (Figure 2). These variables must be recorded not only for reproducibility, but also because they define the contrasts used for candidate screening.

### 4.1. Strain Background Shapes the Initial Component Profile

The components available in a postbiotic preparation depend largely on the strain from which it is produced. Even within the same genus or species, strains may differ substantially in cell envelope architecture, surface protein profiles, exopolysaccharide production, metabolic capacity, and stress tolerance. These biological differences can produce distinct compositional and functional profiles even when cultivation, inactivation, and processing conditions are kept similar [3,36,37]. Comparative analysis of food-derived *Lactiplantibacillus* strains likewise showed strain-dependent differences in oleate-hydratase sequences, predicted protein structures, and conjugated-linoleic-acid production [38].

Such variation is evident in the composition, molecular weight, and branching of exopolysaccharides, as well as in surface-associated proteins and metabolite profiles [39,40,41,42]. These characteristics determine not only the starting composition of the preparation but also how individual components are retained, released, or modified during cultivation and inactivation. Identifying the microorganism at the species level is therefore not enough for meaningful comparison between postbiotics. The strain and, where available, its relevant compositional characteristics should also be documented.

### 4.2. Cultivation Conditions Establish the Pre-Inactivation Composition

Once a strain has been selected, the conditions under which it is grown determine which of its potential components are produced and accumulated before inactivation. Medium composition, substrate, pH, temperature, oxygen availability, and fermentation mode can alter metabolite production, EPS synthesis, cell-surface structures, and microbial stress responses [43,44,45,46]. Studies of *Lactiplantibacillus plantarum* illustrate this influence particularly well: changing the fermentation substrate or culture environment can modify phenolic release, macromolecular structure, and the resulting profile of bioactive metabolites [47,48,49,50,51,52].

The effects of cultivation may persist beyond microbial growth, since the physiological state and composition of the cells can influence what is retained, exposed, or released during inactivation [53,54,55]. Interpretation is especially difficult for soluble components. Compounds detected in the final preparation may have been produced by the microorganism, carried over from the culture medium, or formed through microbial transformation of medium constituents. Cultivation conditions should therefore be reported in sufficient detail, and suitable medium controls should be included wherever residual or transformed medium components could affect compositional analysis [47,48,56,57].

### 4.3. Inactivation Shapes Component Retention, Exposure, Release, and Structure

The composition established during cultivation is further modified when the microorganisms are inactivated. At this stage, the fate of candidate components depends on how strongly the treatment affects the cells. Thermal and non-thermal treatments can alter cell-envelope integrity, membrane permeability, protein conformation, and surface physicochemical properties in different ways and to varying extents [17,58,59]. A component that was previously enclosed within the cell may be released, while an envelope-associated molecule may become more accessible without being detached from the cell surface. Other constituents may remain cell-associated or undergo structural modification and degradation during treatment [60,61,62].

These changes are governed by both the type of treatment and the intensity at which it is applied. More disruptive treatments may increase intracellular release and molecular accessibility, whereas milder treatments may better preserve envelope architecture and cell-associated structures. Postbiotic preparations derived from the same strain can therefore contain different proportions of surface-associated, soluble, and lysis-derived materials, even when their composition before inactivation was similar. The physical form of these materials may also differ, which can affect their recovery, detection, and interaction with host cells [53,59,63,64].

An apparent increase in a component after processing should not, however, be interpreted as evidence that it contributes more strongly to biological activity. An increase may reflect cell disruption, degradation, transformation, or improved analytical accessibility rather than a genuine increase in functional relevance. Meaningful comparison between preparations therefore requires the inactivation method, treatment intensity, and other relevant operating conditions to be described clearly. Subsequent separation, concentration, drying, and storage may further alter component recovery, aggregation, solubility, and accessibility. Cell-envelope fragments, extracellular vesicles, proteins, and polysaccharides may be particularly sensitive because dehydration and reconstitution can change their organization and physical state. Downstream processing conditions should thus be considered alongside cultivation and inactivation when compositional or functional differences among postbiotic preparations are interpreted.

## 5. Experimental Strategies for Narrowing and Prioritizing Candidate Components

**Figure 3:**
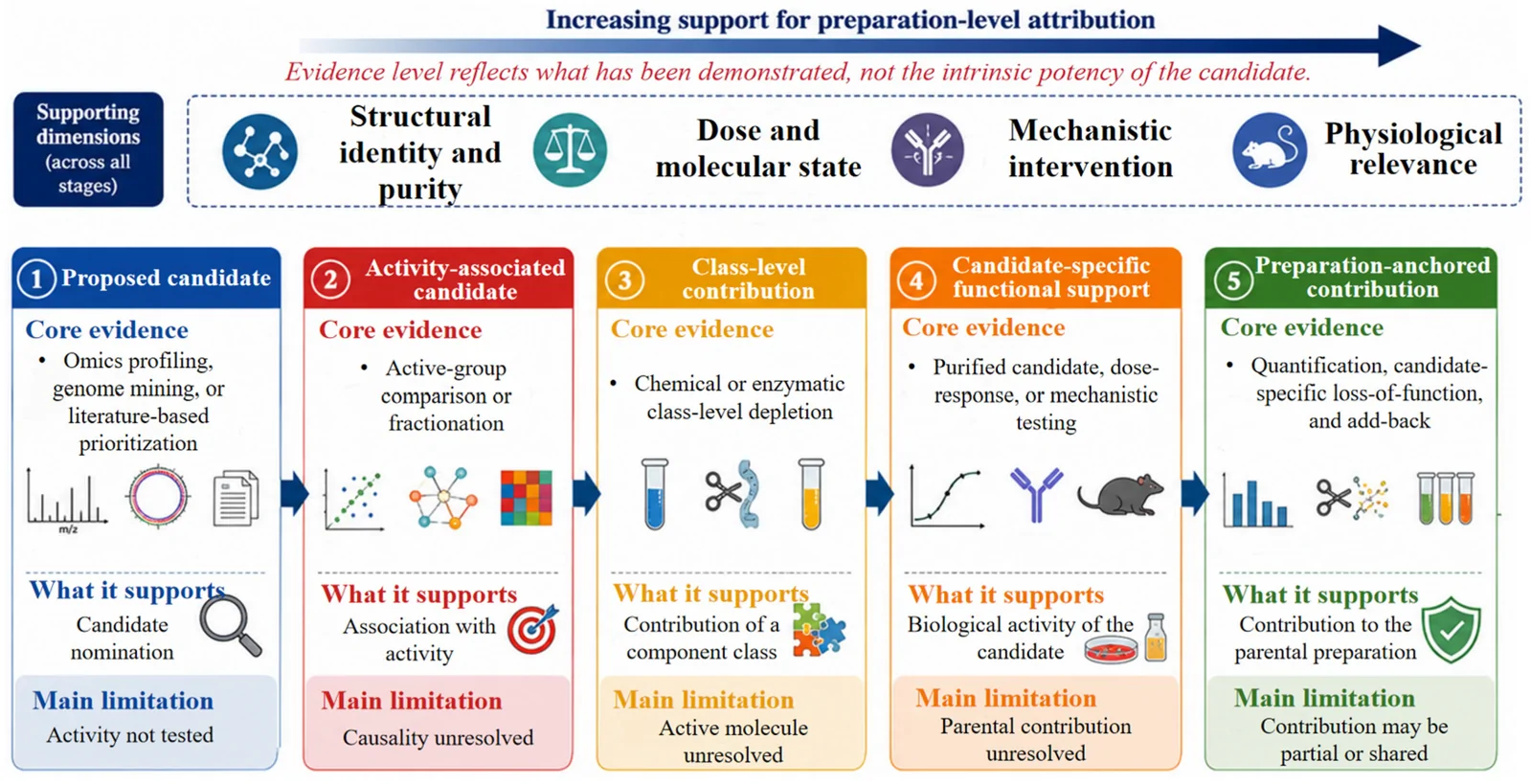
Evidence framework for identifying bioactive components in postbiotic preparations. The framework includes five levels. Candidate components are first proposed through omics profiling, genome mining, or published evidence (Level 1) and then linked to an active sample or fraction (Level 2). Chemical or enzymatic depletion can show whether a broad component class contributes to the activity (Level 3), whereas testing a purified candidate establishes its biological activity but not necessarily its role in the parental preparation (Level 4). Preparation-level attribution requires quantification of the candidate, candidate-specific loss-of-function testing, and recovery of activity after add-back (Level 5). Blue, red, gold, orange, and green represent Levels 1-5, respectively. Structural identity and purity, dose and molecular state, mechanistic intervention, and physiological relevance should be considered at each level. The levels describe the evidence available for a candidate rather than its biological potency.

The search for bioactive components in a complex postbiotic preparation usually begins with a well-defined phenotype and proceeds by progressively excluding less plausible candidates. Activity-based comparisons, fractionation, component-class perturbation, multi-omics profiling, and structural characterization provide different but complementary ways of reducing compositional complexity and selecting a manageable number of candidates for further testing (Figure 3). No single approach is sufficient for this purpose, and the conclusions drawn from each depend on the experimental question, the controls used, and the availability of quantitative data. The following sections examine how these methods can be combined to rank candidate components before their contribution is tested directly.

### 5.1. Phenotype-Guided Comparison of Postbiotic Preparations

Candidate screening is easier to interpret when the preparations being compared show a clear and reproducible difference in biological activity. A representative example is the comparison between live and pasteurized *Akkermansia muciniphila*. In mice fed a high-fat diet, pasteurization preserved or enhanced several metabolic effects, including lower body-weight and fat-mass gain and improved glucose tolerance and insulin sensitivity. This observation shifted attention toward bacterial structures that remained active after heating and supported subsequent investigation of the outer-membrane protein Amuc_1100 [10]. The comparison was informative because the microbial strain remained unchanged while the processing state differed. Amuc_1100 reproduced some of the effects associated with the pasteurized bacterium, but the experiment did not determine how much this protein contributed to the activity of the complete preparation.

The same comparative logic has been applied to preparations exposed to different heating conditions. Itoh et al. [65] and Sun et al. [58], for example, examined postbiotics produced from the same bacterial strains after thermal treatments ranging from 70 to 121 °C for 10 min. Antioxidant and anti-inflammatory activities were largely retained below 100 °C but declined after treatment at 121 °C, with corresponding changes in cell structure and chemical composition. Holding the strain and treatment duration constant made it easier to relate the loss of activity to changes caused by heating. The comparison nevertheless could not distinguish whether the response resulted from the disappearance of an active constituent, the formation of an inhibitory product, or broader changes in the preparation. Likewise, compositional differences following severe heating may reflect molecular release, degradation, or transformation rather than identify the component responsible for the phenotype [58,65].

For phenotype-guided comparison to narrow the candidate pool effectively, the samples should differ in one principal and well-defined feature. Examples include preparations subjected to different inactivation conditions, fractions with contrasting activity obtained from the same preparation, and samples collected before and after a controlled perturbation. The target phenotype and assessment time point should be selected in advance, and the compared samples should be tested at equivalent doses using the same normalization basis. A large and reproducible contrast is generally more useful for candidate discovery than a small difference that reaches statistical significance in only one experiment. Such comparisons help select samples for subsequent fractionation and molecular profiling, although the identity and contribution of the responsible components must still be established in later experiments.

### 5.2. Activity-Guided Fractionation of Candidate Components

Activity-guided fractionation separates a complex postbiotic preparation into simpler fractions and uses a consistent biological assay to determine where the activity is retained. Separation may be based on molecular size, polarity, solubility, charge, or related physicochemical properties. Ultrafiltration, solvent partitioning, precipitation, and chromatography are commonly used for this purpose [66,67]. Each fraction should be compared with the unfractionated preparation using doses traced to the same amount of starting material. Material recovery and biological activity recovery should also be documented throughout the procedure. Otherwise, strong activity in a fraction may result from simple concentration during processing and may not indicate selective enrichment of the constituents that produced the original phenotype.

The isolation of an immunomodulatory phospholipid from *Akkermansia muciniphila* illustrates how this approach can be applied in practice. Bae et al. [68] extracted lipids from bacterial cell pellets and first separated the crude extract by normal-phase chromatography. The resulting fractions were screened for their ability to induce TNF-α production in mouse bone-marrow-derived dendritic cells, after which the active material was separated further by reversed-phase HPLC. Repeating the assay after each separation step localized the response first to a phosphatidylethanolamine-containing fraction and subsequently to a15:0-i15:0 PE. The isolated natural lipid produced a concentration-dependent response, and chemical synthesis, together with the testing of structural analogues, supported its molecular identity and identified structural features required for activity. This work shows how repeated fractionation guided by a reproducible in vitro response can reduce a complex lipid extract to a structurally defined candidate [68].

The separation procedure may, however, change the material as well as divide it into fractions. Solvent, pH, ionic strength, concentration, and disruption of membranes or other supramolecular assemblies can affect the assay response independently of the intended separation. Solvent and process controls are therefore needed, together with an assessment of material lost at each step. Recombining the fractions can also reveal whether the original activity is recovered or depends on interactions among components that were separated during fractionation. Used in this way, activity-guided fractionation can locate activity within a narrower physicochemical range and provide a tractable set of candidates for further analysis. Demonstrating that an individual molecule is necessary for the activity of the original preparation, however, requires subsequent component-specific experiments.

### 5.3. Selective Depletion and Class-Level Contribution Testing

Selective depletion addresses a different question from activity-guided fractionation: whether reducing or disabling a defined constituent or group of constituents changes the activity of the original preparation. Fractionation follows activity into separated fractions, whereas loss-of-function approaches test the effect of removing or disabling a target within the preparation or preventing its production before the preparation is generated. Three approaches should be distinguished: class-level chemical or enzymatic depletion, molecule-specific immunodepletion, and genetic elimination of component production before inactivation. These approaches differ in their specificity and in the conclusions that can be drawn from them.

Chemical or enzymatic treatment generally tests a broad component class. Protease digestion can indicate a contribution from proteins or peptides, whereas neutralization can reveal whether antimicrobial activity depends on acidity. Arrioja-Bretón et al. [69] reported that neutralization removed most of the antimicrobial activity from several lactic-acid-bacterial cell-free supernatants. In one *Lactobacillus plantarum* supernatant, however, the remaining activity disappeared after proteinase K treatment, pointing to an additional proteinaceous factor. Similarly, proteinase K reduced the antibacterial activity of the *L. plantarum* NIBR97 supernatant against *Salmonella* by approximately 50% [70]. These studies help identify active component classes but do not reveal the individual molecules involved. Moreover, because the tested supernatants do not necessarily meet the ISAPP definition, they serve mainly as methodological examples.

Immunodepletion offers a more specific test. Yan et al. used antibodies to remove p40 and p75 from the *Lacticaseibacillus rhamnosus* GG-conditioned medium. Their removal reduced Akt activation and reversed the protection against cytokine-induced epithelial-cell apoptosis [71]. This provides stronger support for individual candidates than class-level protease treatment, although the study remains a mechanistic analogue rather than direct postbiotic evidence. Genetic elimination prevents the microorganism from producing the candidate before the preparation is made. Mohamadzadeh et al. deleted a gene required for lipoteichoic acid biosynthesis in *Lactobacillus acidophilus* NCFM and showed that the LTA-deficient mutant differed from the parental strain in its immunological effects [72]. Because viable bacteria were tested, this study illustrates the approach but does not provide direct postbiotic evidence. For postbiotic attribution, wild-type and mutant strains should be cultured and inactivated under the same conditions, with genetic complementation included where possible.

All three approaches require evidence that the intended target was reduced or eliminated without major changes to the rest of the preparation. Appropriate controls include mock or reagent treatments, nonspecific antibody controls, and matched wild-type strains. A decline in activity does not show that the target is solely responsible, because incomplete depletion, off-target effects, genetic changes elsewhere in the cell, and interactions among components may also affect the response. The evidence is strongest when loss of activity is followed by reconstitution at a concentration measured in the original preparation.

### 5.4. Multi-Omics Identification and Association-Based Prioritization

Once activity has been localized to particular samples, fractions, or component classes, multi-omics profiling can help determine which molecules warrant further investigation. The choice of platform should reflect the physicochemical nature of the suspected candidates. Metabolomics is most useful for small-molecule candidates, including organic acids, amino acid derivatives, short peptides, amines, nucleotides, and tryptophan-related metabolites [73,74]. Lipidomics is better suited to membrane lipids and glycolipids, while proteomic and surface-proteomic methods can identify extracellular, surface-associated, or lysis-exposed proteins [41,75,76]. Polysaccharides require a different analytical route because molecular weight, monosaccharide composition, branching, linkage pattern, and higher-order structure may be more informative than abundance alone [45]. Studies of fermented food materials also show that transcriptomic and metabolomic profiling can relate fermentation-associated changes to cellular, animal, or gut–liver phenotypes [77,78,79,80]. Such relationships can prioritize pathways and molecular features, but they do not by themselves identify microbial effectors.

Molecular features can then be ranked according to how closely they follow the phenotype of interest. Fonseca et al. [81], for example, compared the metabolomic profiles of probiotic culture supernatants that differed in immunoregulatory activity. Features that discriminated the more active supernatants were identified, and pathway analysis pointed to tryptophan metabolism as a possible source of immunoregulatory molecules. The analysis reduced a complex dataset to a smaller group of candidate metabolites and pathways. However, whether these features contributed to the observed response remains uncertain: most of the features were only putatively annotated and were neither selectively depleted from nor individually added back to the source material.

Correlation, regression, and network-based analyses offer similar ways to identify molecules that repeatedly vary with inflammatory, barrier-related, metabolic, or other functional readouts [82,83]. These methods are most useful for setting priorities, since molecules may co-vary because they share a metabolic pathway, respond similarly to processing, or are affected by the same batch-related factor. A feature detected only through molecular profiling should remain a candidate; repeated association with an active sample or fraction provides stronger grounds for prioritization but still falls short of functional attribution. That step requires targeted experiments conducted in relation to parental preparation.

### 5.5. Structural Characterization of Prioritized Candidates

After the candidate pool has been narrowed, the selected components must be characterized sufficiently to determine what material is actually being tested. This includes its molecular identity and purity, as well as the physical form in which it occurs. A molecular name alone is often insufficient, particularly for heterogeneous candidates such as exopolysaccharides, peptidoglycan fragments, lipoteichoic acids, membrane lipids, and microbial proteins. Members of the same nominal class may differ in molecular weight, substitution pattern, acyl-chain composition, conformation, aggregation state, or association with other cellular structures, and these differences can alter their biological effects [12].

The analytical requirements vary with the type and complexity of the candidate. MS, MS/MS, and NMR can support molecular identity and structural assignment, whereas chromatographic and light-scattering methods are useful for describing molecular-weight distributions, sample dispersity, and compositional heterogeneity. Spectroscopic methods, including FTIR and Raman spectroscopy, can provide complementary information on functional groups and chemical composition, while microscopy may help assess cellular morphology, membrane structures, or particle organization [5,84,85,86]. Complex candidates usually require more than one analytical technique because each method resolves only part of the structure. Claims about molecular identity should therefore be supported by complementary measurements and by evidence that contaminants or co-purified materials are unlikely to account for the observed activity.

Adequate characterization makes it possible to quantify a candidate, compare it across preparations, and reproduce its testing in subsequent experiments. It also helps distinguish genuine structural variation from differences caused by extraction, purification, or storage. Characterization alone, however, cannot show whether the component contributes to the biological activity of its source preparation. Table 2 summarizes the roles, design requirements, and interpretive limits of phenotype-guided comparison, activity-guided fractionation, class-level depletion, multi-omics prioritization, and structural characterization. Together, these strategies provide a practical route from a complex preparation to a smaller set of sufficiently defined candidates, which can then enter the direct functional tests described in the next section.

## 6. Functional Validation and Evidence Appraisal of Prioritized Candidates

### 6.1. Candidate-Specific Depletion and Reconstitution

Once a candidate has been prioritized and adequately characterized, the next question is whether it makes a measurable contribution to the activity of the original postbiotic preparation. The most direct design includes the untreated preparation, a mock-treated preparation, the candidate-depleted preparation, and the depleted preparation after the candidate has been added back. Depletion efficiency must be quantified, and possible changes in non-target constituents should also be monitored. A reduction in activity following selective depletion, together with recovery after the candidate is restored at its endogenous concentration, provides substantially stronger evidence of contribution than testing the purified candidate alone.

Few postbiotic studies have completed this full depletion–reconstitution sequence. Frequently cited candidates, including Amuc_1100, P9, and lipoteichoic acid, have reproduced particular effects reported for their source bacteria or preparations [10,76,87]. These experiments demonstrate that the isolated candidates are biologically active under the conditions tested, but they do not show how much each candidate contributes to the corresponding preparation-level phenotype. That question remains unresolved because the candidate was not selectively removed from the source preparation before being restored. The degree of recovery in an add-back experiment must also be considered alongside the efficiency of depletion, the activity of the untreated preparation, and the dose and physical form of the reintroduced candidate. Partial recovery may indicate incomplete restoration, altered molecular presentation, or a response that depends on several components acting together.

### 6.2. Dose–Response Relationships and Preparation-Relevant Exposure

Dose–response experiments are easier to interpret when the tested dose of a purified candidate is compared with its measured content in the original preparation. The key question is not only whether the candidate becomes active as its dose increases, but whether the preparation contains enough of it to produce the observed response. Answering this question requires targeted or absolute quantification, followed by testing at concentrations equivalent to those delivered by the preparation.

For example, Yu et al. [88] tested an exopolysaccharide isolated from *Lacticaseibacillus paracasei* at three doses in mice infected with *Helicobacter pylori*. Higher doses generally produced greater improvements in gastric inflammation and microbiota-related outcomes. These results support the activity of the purified EPS, but they do not show whether a corresponding bacterial preparation would deliver enough EPS to produce the same effects. Liu et al. [25], in contrast, found that the immunomodulatory activity of lipoteichoic acid from *Limosilactobacillus reuteri* L1 varied with both dose and inflammatory status in RAW264.7 cells and mice. This result shows that immune-active components may behave differently under normal and inflammatory conditions and may not produce a simple linear dose–response relationship.

The route of administration, molecular form, and surrounding matrix also affect the dose that reaches the biological target. A purified molecule in solution may behave differently when it remains attached to the cell surface, forms aggregates, or is incorporated into a food matrix. Digestion can further change its structure and availability. A reproducible dose–response relationship supports the biological relevance of a candidate, particularly when activity occurs near the concentration present in the preparation. It does not, however, replace direct testing of the candidate’s contribution to the activity of the original preparation.

### 6.3. Mechanistic Intervention and Target Engagement

Mechanistic support should distinguish pathway association from pathway dependence. Changes in receptor abundance, phosphorylation status, or downstream gene expression indicate that a signaling pathway is engaged, but they do not establish that the pathway is required for the candidate-induced phenotype. Stronger evidence is obtained when inhibition, gene silencing, receptor deletion, or loss-of-function mutation attenuates the effect, particularly when direct candidate–target interaction is also demonstrated.

Several studies illustrate this distinction. Bae et al. [68] showed that the immunomodulatory activity of the *Akkermansia muciniphila* phospholipid a15:0-i15:0 PE required the TLR2-TLR1 heterodimer, and structural analogues were used to define the molecular features required for signaling. Gao et al. [89] provided stronger pathway-level evidence by showing that the protective effects of the peptidoglycan hydrolase LPH were lost in NOD2-deficient mice, linking enzymatic generation of MDP to NOD2-dependent gut protection. Similarly, Amuc_1409 was shown to interact with E-cadherin, promote dissociation of the E-cadherin/β-catenin complex, and activate Wnt/β-catenin signaling; deletion of E-cadherin in intestinal stem cells abolished its regenerative effects [90].

These studies show that mechanism is better supported when receptor engagement and phenotypic dependence are demonstrated directly. However, strong target-level evidence does not by itself establish that the candidate accounts for the activity of the parental postbiotic preparation. Mechanistic intervention should therefore be interpreted together with preparation-level depletion, dose relevance, and in vivo evidence [91,92,93,94].

### 6.4. Cross-Model Validation and Physiological Relevance

Evidence becomes more convincing when the same well-characterized candidate produces related effects across models of increasing biological complexity. Cell-free or cell-based assays can reveal direct molecular and cellular responses, while animal studies additionally account for delivery, stability, tissue exposure, metabolism, and interactions with the intestinal environment. The endpoints used across these models should remain biologically connected so that the results can be interpreted as evidence for the same underlying effect.

The protein HM0539 from *Lacticaseibacillus rhamnosus* GG provides one example. Purified HM0539 increased the expression of mucins and tight-junction proteins and limited barrier disruption induced by LPS or TNF-α in Caco-2 monolayers. Oral administration of the same protein subsequently improved intestinal barrier-related outcomes in neonatal rats, as well as in mouse models of DSS-induced colitis and LPS/GalN-induced injury [95]. Lactocepin illustrates a different form of validation. The purified enzyme degraded the pro-inflammatory chemokine IP-10, and disruption of the lactocepin-encoding prtP gene reduced the anti-inflammatory activity of the source strain in a mouse model of colitis [96].

Consistent biochemical, cellular, and animal findings increase confidence that a candidate can act under physiologically relevant conditions. They also help identify effects that are limited to a particular assay or experimental model. Nevertheless, activity across several models does not establish the candidate’s contribution to a defined postbiotic preparation. The lactocepin study, for example, demonstrated loss of function in a live bacterial strain rather than in an inactivated preparation. Preparation-level attribution still requires measurement of the candidate in the postbiotic, followed where possible by selective depletion and add-back at a relevant dose.

### 6.5. Integrating Evidence for Functional Attribution

No single experiment can fully determine whether a candidate accounts for part of a postbiotic preparation’s activity. Depletion and add-back experiments test its contribution within the preparation, while dose–response studies determine whether it remains active at the amount and in the physical form actually present. Targeted pathway interventions establish whether the proposed receptor or signaling route is required, and cross-model studies examine whether the effect persists under more physiologically relevant conditions. Together, these approaches address contribution, dose relevance, mechanism, and biological validity.

To assess how far current studies meet these requirements, representative primary studies were evaluated for preparation definition, candidate identification and quantification, selective depletion, reconstitution, dose relevance, mechanistic intervention, and in vivo support (Table 3). Five evidence categories were used: proposed candidate, activity-associated candidate, class-level contribution, candidate-specific functional support, and preparation-anchored contribution. These categories indicate what has been demonstrated experimentally; they do not rank the potency or potential importance of the candidates.

Among the 15 studies assessed, one reported a proposed candidate, three identified activity-associated candidates, one supported a component class, and ten provided candidate-specific functional support. None met the criteria for preparation-anchored contribution. Across these studies, purified proteins, lipids, cell-wall components, polysaccharides, and vesicle-associated systems often produced direct biological effects, engaged a proposed receptor, or remained active in vivo. However, few studies quantified the candidate in the source preparation and then combined selective depletion with add-back at the corresponding dose. The evidence therefore supports the activity and mechanistic plausibility of many candidates, but rarely shows how much they contribute to the phenotype of the complete preparation.

Some preparation-level effects are also unlikely to arise from a single constituent. Residual activity after class-level perturbation, incomplete loss following depletion, or activity detected in several fractions may point to contributions from multiple components, but these patterns are not evidence of synergy on their own. If one candidate explains only part of the phenotype, defined combinations should be tested at preparation-relevant ratios and evaluated against an appropriate additive model.

## 7. Current Limitations and Future Directions for Functional Attribution

Research has identified numerous microbial proteins, polysaccharides, cell-wall components, lipids, metabolites, and vesicle-associated factors with biological activity. Yet, as Table 3 shows, progress in identifying candidates has outpaced efforts to determine their contribution to complete postbiotic preparations. The main difficulty is no longer finding potentially active molecules, but establishing how much of each candidate is present, in what physical form it occurs, and whether it contributes measurably to the preparation-level phenotype.

### 7.1. Evidence Gaps Between Candidate Activity and Preparation-Level Attribution

Candidates selected through comparative omics or activity-guided fractionation are often tested as purified or recombinant molecules. Amuc_1100, lipoteichoic acid associated with heat-killed *Lacticaseibacillus paracasei* D3-5, and the *Akkermansia muciniphila* phospholipid a15:0-i15:0 PE all reproduced particular effects in cellular or animal models [10,68,87]. These studies confirm that the candidates can be active, but none combined their selective removal from the source material with add-back at the amount present in the preparation. Their contribution to the complete preparation therefore remains unresolved.

Two recurring gaps are evident in Table 3. First, candidate concentrations in the source preparation are rarely measured. Second, purification may change molecular organization, accessibility, or effective exposure, especially for components associated with membranes, cell walls, or extracellular vesicles. Both gaps are closely related to how the preparation is produced and handled. The strain, culture medium, and growth conditions establish its starting composition, while inactivation, downstream processing, storage conditions and duration, and the final matrix determine how much of a candidate is retained, released, transformed, or accessible. These variables should therefore be reported together with the basis used to normalize preparations, such as dry mass, cell equivalents, or a relevant compositional marker. When candidate abundance is measured, the amount delivered at the tested preparation dose can be compared directly with the purified-candidate dose used in cells or animals. Without this link, differences in activity may reflect unequal candidate exposure rather than differences in functional contribution. Candidate quantification should therefore provide the basis for subsequent depletion and add-back experiments at preparation-relevant doses.

### 7.2. Structural Heterogeneity and Experimental Context Contribute to Inconsistent Findings

Components grouped under the same name may produce different or even opposing effects across studies. This does not always represent a true contradiction. Lipoteichoic acids, exopolysaccharides, and extracellular vesicles are heterogeneous materials whose activity can change with molecular structure, purity, physical state, dose, and the model in which they are tested. Findings for one preparation or molecule should therefore not be extended automatically to an entire component class. This heterogeneity also determines which analytical and functional approaches are informative for each component class.

Lipoteichoic acid illustrates this problem particularly well. LTAs from different species and strains vary in backbone composition, chain length, glycosylation, D-alanylation, and lipid-anchor structure. Modifications such as deacylation can change receptor recognition and immune activity, while incomplete purification may introduce co-isolated substances that affect the measured response [106,107,108]. LTA cannot therefore be classified universally as pro- or anti-inflammatory. Its activity must be interpreted in relation to its microbial source, structural features, purity, dose, and experimental model. Hydrophobic-interaction chromatography, followed by NMR and MS, can help define the purity and structural features of LTA. PGN requires a different approach: digestion into muropeptides followed by HPLC or LC-MS can reveal peptide-stem composition, cross-linking, and other structural modifications [106,107,108,109]. Their functional contribution may then be examined by candidate-directed depletion, class-specific enzymatic treatment, or matched changes in microbial biosynthesis where feasible. Such interventions require careful controls because they may also alter other cell-envelope components.

Exopolysaccharides vary in monosaccharide composition, glycosidic linkages, molecular weight, branching, conformation, charge, and chemical substitution, all of which may influence their physicochemical and biological properties. Residual proteins, nucleic acids, or cell-wall materials can further complicate interpretation [110,111]. Total carbohydrate content alone is therefore insufficient for comparing EPS preparations. Molecular-weight analysis, monosaccharide and linkage analysis, and NMR provide complementary information on their composition and structure. A primary study of EPS from *Bacillus licheniformis* AG-06 illustrates this combined approach: the polymer was purified and characterized for molecular mass and composition before its functional properties were examined [112]. When a suitable glycosidase or producer mutant is available, enzymatic treatment or loss of EPS production can provide functional evidence, although matched inactivated preparations are needed to establish direct postbiotic relevance.

Extracellular vesicles are even more complex because the intact particles carry mixtures of lipids, proteins, nucleic acids, metabolites, and cell-wall-associated ligands. Detecting a cargo molecule does not show that it is responsible for vesicle activity. Comparisons across studies should therefore be based on adequately characterized materials tested at comparable doses and under relevant model conditions, rather than assuming that all members of a component class are functionally interchangeable. Vesicle studies therefore require separation from cells, debris, and soluble material using complementary size- and density-based methods. Particle concentration and size, electron microscopy, and biochemical cargo analysis can then define the material being tested [113]. Comparing vesicle-depleted preparations with particle-matched add-back would provide stronger evidence of their contribution than testing isolated vesicles alone.

Candidates that can be defined as individual molecules require a more targeted approach, but their analysis should still remain linked to the parental preparation. Proteins can be quantified by targeted LC-MS/MS or immunoassays and tested by molecule-specific immunodepletion and recombinant add-back [5]. Metabolites can be quantified by targeted LC-MS or GC-MS with authentic or isotope-labelled standards, allowing purified compounds or defined mixtures to be tested at concentrations measured in the preparation. For lipids, class-specific extraction and LC-MS/MS lipidomics can resolve individual molecular species. Phospholipase treatment can provide class-level evidence, whereas synthetic-lipid testing can confirm the activity of a defined structure [5,68].

### 7.3. Limitations of Single-Candidate Attribution in Multicomponent Preparations

A postbiotic may contain several active component classes, making it unlikely that one candidate will explain every aspect of its biological effect. Class-level perturbation studies offer indirect evidence of such shared contributions. Neutralization, for example, removed most of the antimicrobial activity from several lactic-acid-bacterial cell-free supernatants, while proteinase K abolished the activity that remained in some *Lactiplantibacillus plantarum* samples. This pattern points to contributions from both organic acids and proteinaceous compounds [69]. Proteinase K reduced the antimicrobial activity of the *L. plantarum* NIBR97 supernatant by only about 50%, and a <3 kDa fraction from *Lacticaseibacillus paracasei* CH88 retained some activity after protease treatment [70]. Although these supernatant studies do not necessarily involve preparations that meet the ISAPP definition of a postbiotic, they illustrate how residual activity can indicate more than one active component class. They do not, however, prove that the components act synergistically.

Partial loss after depletion or incomplete recovery after adding back a single candidate should prompt the testing of a limited number of defined component combinations. Fraction recombination and combination add-back experiments can determine whether the original activity can be recovered, while factorial or dose–matrix designs can distinguish additive, synergistic, and antagonistic responses. The candidates should be structurally characterized and quantified, and their test doses and ratios should approximate those measured in the original preparation.

A postbiotic preparation may behave differently after it is added to a food. Proteins, fats, and carbohydrates in the food may bind or entrap microbial components and change their release during digestion. Heating after the postbiotic has been added, as well as subsequent storage, may also alter proteins, metabolites, lipids, and vesicles [1,4]. The finished food should therefore be tested after normal processing and storage, using the amount provided in one serving [4,14]. When an intestinal effect is expected, simulated digestion can help determine how much of a candidate component remains accessible. The amount that can be added may also be limited if it makes the product too acidic or bitter or changes its aroma, colour, or texture [4]. Evidence obtained with the postbiotic ingredient alone may therefore not fully represent its activity in the final food.

### 7.4. Analytical and Statistical Challenges in Postbiotic Research

Detailed characterization of postbiotic components often requires advanced tools such as high-resolution mass spectrometry, MS/MS, NMR, chromatography, and imaging methods [5,84,85,86]. These methods are costly and require trained staff, which can limit sample sizes, the inclusion of independent production batches, and replication across laboratories. Results may also differ because laboratories use different extraction methods, instruments, reference libraries, and data-processing procedures. One practical approach is to screen samples first through phenotype-guided comparison, fractionation, and class-level assays, and then apply advanced structural analysis only to the most promising candidates. Shared facilities, reference materials, standard protocols, and clear reporting of methods and raw data can further improve access and comparability.

Variation caused by microbial source and processing also requires careful experimental design. Studies should include independent production batches rather than technical replicates alone and should report cultivation, inactivation, storage, and analytical conditions. Randomization, quality-control samples, adequate sample sizes, and statistical models that account for batch effects can help reduce false associations. Omics studies should also correct for multiple testing, report effect sizes, and use cross-validation or independent validation where possible [82,83]. Statistical analysis can help rank candidates, but it cannot correct a poorly designed study or prove functional contribution without targeted experiments. Reliable conclusions therefore require sound experimental design, clear reporting, and biological validation.

## 8. Conclusions

This review distinguishes the activity of an isolated microbial component from its contribution to a postbiotic preparation. Omics profiling, association analysis, fractionation, class-directed perturbation, and purified-candidate testing can identify and evaluate bioactive candidates, but preparation-level contribution is more convincingly supported by candidate quantification, selective depletion, and reconstitution at a preparation-relevant dose. Mechanistic and in vivo evidence strengthen interpretation but do not replace this link to the parental preparation. Because proteins, polysaccharides, cell-wall components, lipids, metabolites, and extracellular vesicles differ in structure and physical state, their evaluation requires component-specific methods. Defined combinations should be examined when a single candidate explains only part of the parental phenotype. Linking candidate abundance and structural state to biological activity may also support batch consistency, potency assessment, and dose interpretation in product development and human studies.

## Figures and Tables

**Table 1:** Major source categories of candidate bioactive components in postbiotic preparations.

Component Category	Main Microbial Source or Location	Representative Components	Main Consideration for Functional Attribution
Cell wall and surface-associated structures	Cell wall, cytoplasmic membrane, surface layers and appendages	PGN, WTA, LTA, surface proteins, adhesins, pili-associated proteins, membrane lipids and glycolipids	Activity may depend on molecular structure, surface exposure and association with other envelope components
Extracellular or soluble factors	Culture medium and extracellular matrix	SCFAs, organic acids, tryptophan-derived metabolites, vitamins, peptides, bacteriocins and EPS	Measured activity may be influenced by concentration, medium background, extraction, purity and the amount retained in the final preparation
Intracellular or lysis-derived constituents	Cytoplasm and nucleoid; released following membrane damage or cell lysis	Proteins, enzymes, intracellular peptides, nucleic acid fragments and cytoplasmic metabolites	Release and accessibility depend strongly on processing, and increased abundance after lysis does not establish functional contribution

Note: The categories describe the principal source of each component and are not mutually exclusive. Processing may redistribute components between cell-associated and soluble states. Extracellular vesicles represent a particle-associated form that may carry lipids, proteins, nucleic acids, metabolites and cell-wall-associated molecules.

**Table 2:** Strategies for narrowing and prioritizing candidate components in postbiotic preparations.

Approach	Main Question	Essential Considerations	Interpretive Value	Main Limitation
Phenotype-guided comparison	Which preparations provide a clear functional contrast?	Predefined phenotype; Matched strain, processing, dose, and matrix where possible	Selects informative samples for subsequent comparison	Does not link the phenotype to a specific component
Activity-guided fractionation	In which fraction is activity retained or enriched?	Unfractionated control; Equivalent-dose testing; Material and activity recovery	Narrows activity to a fraction or physicochemical range	Activity may reflect concentration, co-fractionation, or loss of interactions
Component-class depletion	Does perturbing a component class reduce activity?	Verified depletion; Mock treatment; Control of non-target losses	Supports contribution at the component-class level	Broad treatments may alter other components or the physical state of the preparation
Multi-omics and association analysis	Which molecular features distinguish active samples or track with the phenotype?	Informative sample comparison; Suitable blanks; Reliable annotation and statistical control	Identifies candidates and associated candidates	Differential abundance or correlation does not establish functional contribution
Structural characterization	What is the identity and structural state of the prioritized candidate?	Component-specific methods; Purity and heterogeneity assessment	Produces a defined candidate suitable for direct testing	Structural definition alone does not demonstrate a role in the parental preparation

Note: These approaches are not necessarily applied in a fixed sequence. Their methods should be adapted to the component class, and none alone establishes functional contribution to the parental preparation.

**Table 3:** Evidence appraisal of representative candidate bioactive components in postbiotic preparations and related mechanistic studies.

Candidate	Source	Meets ISAPP Postbiotic Definition	Supporting Evidence	Evidence-Source Group	Current Evidence Status	Key Missing Evidence	Reference
Amuc_1100	Pasteurized *Akkermansia muciniphila*	Yes	Purified protein; TLR2-related activity; Mouse validation	Mixed: direct postbiotic evidence and methodological/mechanistic analogue	Candidate-specific functional support	No candidate-specific depletion or parent-matched add-back	[10]
P9	Active *A. muciniphila* cell-free supernatant	No	Activity-guided purification; Recombinant protein; ICAM-2-related mechanism; Mouse validation	Methodological/mechanistic analogue	Candidate-specific functional support	No P9-specific depletion; Exposure not matched to the parental	[97]
a15:0-i15:0 PE	*A. muciniphila* lipid extract	No	Activity-guided purification; Synthetic confirmation; TLR2-TLR1 dependence	Methodological/mechanistic analogue	Candidate-specific functional support	No preparation-level depletion or direct in vivo test of the lipid	[68]
LTA	Heat-killed *Lacticaseibacillus paracasei* D3-5	Yes	Purified LTA reproduced barrier and anti-inflammatory effects in vivo	Mixed: direct postbiotic evidence and postbiotic-derived component evidence	Candidate-specific functional support	No LTA-specific depletion or parent-equivalent dosing	[87]
Muramyl dipeptide	Defined peptidoglycan ligand; no parental preparation	No	Defined molecule; colitis-model activity; NOD2- and autophagy-related changes	Methodological/mechanistic analogue	Candidate-specific functional support	Not linked to a defined postbiotic preparation	[98]
LPH-MDP module	*Lactobacillus* secretomes, lysates, and supernatants	No	Active-site mutants; MDP generation; *Nod2* knockout; mouse validation	Methodological/mechanistic analogue	Candidate-specific functional support	No depletion or reconstitution in a defined postbiotic preparation	[89]
*A. muciniphila* extracellular vesicles	Purified bacterial vesicles	No	Barrier-related and metabolic effects in mice	Methodological/mechanistic analogue	Candidate-specific functional support	Active cargo and contribution to the whole-bacterium preparation remain unresolved	[99]
Acidic and proteinaceous components	Lactic-acid-bacterial cell-free supernatants	No	Neutralization and proteinase K treatment reduced antimicrobial activity	Methodological/mechanistic analogue	Class-level contribution	Broad perturbation; individual molecules not identified	[69]
Immunoregulatory metabolomic features	Probiotic culture supernatants	No	Molecular features and pathways associated with active supernatants	Methodological/mechanistic analogue	Activity-associated candidate	Tentative identity; no absolute quantification or targeted testing	[81]
p40 and p75	Soluble fraction of *Lacticaseibacillus rhamnosus* GG-fermented milk	No	Immunodepletion abolished epithelial protection; mouse validation	Methodological/mechanistic analogue	Candidate-specific functional support	No inactivated preparation or add-back	[100]
Soluble polysaccharides (SPS)	Heat-inactivated *Bifidobacterium adolescentis* ATCC 15703	Yes	Crude-component fractionation; SPS activity in colonoids; parental preparation validated in mice	Mixed: direct postbiotic and postbiotic-derived component evidence	Activity-associated candidate	No SPS-specific depletion, preparation-matched add-back, or in vivo SPS test	[101]
Surface-associated EPS	Live *Bifidobacterium breve* UCC2003 and EPS-deficient variants	No	Genetic disruption; immune modulation and pathogen protection in mice	Methodological/mechanistic analogue	Candidate-specific functional support	No matched inactivated preparations	[102]
Cell-wall-associated factors, including proposed LTA	Inactivated *Bacillus coagulans* GBI-30, 6086	Not established	Inactivated cells activated human immune cells and altered cytokine responses in vitro	Methodological/mechanistic analogue	Proposed candidate	Candidate components not directly tested	[103]
β-Glucan-containing cell-wall material	*Kluyveromyces marxianus* and *Saccharomyces boulardii*	No	Dendritic-cell cytokine responses; partial inhibition by Dectin-1 blockade	Methodological/mechanistic analogue	Candidate-specific functional support	No β-glucan-specific depletion or host validation	[104]
Fermented-formula components	Heat-treated formula fermented with *Bifidobacterium breve* C50 and *Streptococcus thermophilus* 065	Yes	Clinical evaluation; reduced faecal calprotectin and increased secretory IgA	Direct postbiotic evidence	Activity-associated candidate	Components not identified or tested separately	[105]

Note: The assessment of whether the ISAPP postbiotic definition is met applies to the source preparation rather than to the isolated candidate component. “Yes” indicates that the study tested a defined inactivated microbial preparation with a demonstrated host benefit; “No” indicates that the experimental material did not constitute a qualifying inactivated preparation; and “Not established” indicates that preparation-level information or host-benefit evidence was insufficient. Evidence-source groups comprise direct postbiotic evidence, postbiotic-derived component evidence, and methodological/mechanistic analogues, as defined in Section 2.1. “Mixed” indicates that more than one evidence-source group was represented in the same study. Current evidence status was assigned using five categories: proposed candidate, activity-associated candidate, class-level contribution, candidate-specific functional support, and preparation-anchored contribution. These categories describe what has been demonstrated experimentally and do not rank the biological potency or clinical importance of the candidates. Abbreviations: ISAPP, International Scientific Association for Probiotics and Prebiotics; TLR, Toll-like receptor; ICAM-2, intercellular adhesion molecule 2; PE, phosphatidylethanolamine; LTA, lipoteichoic acid; MDP, muramyl dipeptide; EPS, exopolysaccharide; SPS, soluble polysaccharides; IgA, immunoglobulin A. The categories describe the current type of evidence rather than molecular potency. Candidate-specific activity does not establish contribution to the parental preparation. None of the studies listed provided preparation-anchored evidence combining candidate quantification, selective depletion, preparation-relevant add-back, and in vivo confirmation.

## Data Availability

No new data was used for the research described in the article. The information used to construct Table 3 was extracted from the cited primary studies and classified according to the study-selection and evidence-appraisal criteria described in Section Literature Search and Study Selection and Section 2.1. The information supporting the appraisal is summarized in Table 3 and is available in the cited publications.
